# AGR2: The Covert Driver and New Dawn of Hepatobiliary and Pancreatic Cancer Treatment

**DOI:** 10.3390/biom14070743

**Published:** 2024-06-23

**Authors:** Shen Qu, Weili Jia, Ye Nie, Wen Shi, Chao Chen, Zihao Zhao, Wenjie Song

**Affiliations:** 1 Xi’an Medical University, Xi’an 710021, China; 18691390385@163.com (S.Q.); wileychia@msn.com (W.J.); 17839970898@163.com (W.S.); chenchao076@163.com (C.C.); 2 Department of Hepatobiliary Surgery, Xijing Hospital, Fourth Military Medical University, Xi’an 710032, China; nieyy1022@163.com (Y.N.); zihaozhao927@163.com (Z.Z.)

**Keywords:** AGR2, hepatobiliary and pancreatic cancer, endoplasmic reticulum stress (ERS), immunotherapy, cancer invasion and prognosis

## Abstract

The anterior gradient protein 2 (AGR2) plays a crucial role in facilitating the formation of protein disulfide bonds within the endoplasmic reticulum (ER). Research suggests that AGR2 can function as an oncogene, with its heightened expression linked to the advancement of hepatobiliary and pancreatic cancers through invasion and metastasis. Notably, AGR2 not only serves as a pro-oncogenic agent but also as a downstream targeting protein, indirectly fostering cancer progression. This comprehensive review delves into the established functions and expression patterns of AGR2, emphasizing its pivotal role in cancer progression, particularly in hepatobiliary and pancreatic malignancies. Furthermore, AGR2 emerges as a potential cancer prognostic marker and a promising target for immunotherapy, offering novel avenues for the treatment of hepatobiliary and pancreatic cancers and enhancing patient outcomes.

## 1. Introduction

Hepatobiliary and pancreatic cancer pose significant challenges among gastrointestinal malignancies. Hepatocellular carcinoma (HCC) stands out as the primary form of liver cancer [1], ranking as the sixth-most prevalent cancer globally and the third-leading cause of cancer-related fatalities [2]. The mortality rates are alarmingly high [3], with projections from the World Health Organization indicating a stark increase in new cases and deaths by 2040 [4]. Similarly, prognosis for bile duct and pancreatic cancers remains complex. Pancreatic cancer is a major contributor to cancer-related deaths worldwide, while the mortality rate for bile duct cancer has seen a notable uptick [5]. Detection often transpires at advanced stages, limiting treatment options and efficacy [6]. Most of the cancers are already at an advanced stage when detected, resulting in patients missing the best opportunity for treatment [1,7]. Late-stage patients commonly undergo chemotherapy or targeted therapy, though results are often unsatisfactory [8,9].

The emergence of drug resistance within tumor cells significantly undermines treatment efficacy, with AGR2 implicated in promoting cancer progression and fostering drug resistance [10,11]. Specifically, AGR2 plays a dual role in cancer progression. On the one hand, it promotes cancer cell proliferation, invasion, and metastasis through its various functions. On the other hand, AGR2 is overexpressed, which reduces the vulnerability of cancer cells and contributes to the development of drug resistance [12]. These effects have been validated in intrahepatic cholangiocarcinoma (ICC) [13], fibrolamellar carcinoma of the liver (FLC) [14], metastatic hepatocellular cancer [15], and pancreatic cancer [16]. In recent years, immunotherapy has gained prominence as a treatment modality for cancer. AGR2 has shown promise as a prognostic marker in prostate and pancreatic cancers, guiding immunotherapy strategies [17,18]. The mounting evidence underscores the importance of AGR2 in cancer immunotherapy, particularly in hepatobiliary and pancreatic cancers, suggesting its potential as a valuable target for novel therapeutic approaches [12,16,19,20]. Therefore, understanding the expression patterns of AGR2 in hepatobiliary and pancreatic cancers carries significant implications for exploring new avenues in cancer immunotherapy. By identifying reliable molecular biomarkers, we can improve cancer detection, monitoring, prognosis prediction, and gain insights into tumor development mechanisms. These efforts are important for advancing personalized cancer treatment strategies.

## 2. Background of AGR2

AGR2, also referred to as anterior gradient protein 2, PDIA17, XAG-2, or hAG-2, is a homolog of the secretory adenosine protein XAG-2. It belongs to the protein disulfide isomerase (PDI) family, serving as an ER retention protein. AGR2 plays a crucial stabilizing role and is recognized as the 17th member of the PDI family [21]. It typically functions in the tumor microenvironment (TME), promoting the growth and invasion of cancer cells [22], while also contributing to the proliferation and metastasis of cancer cells across various cancer types [23]. Like AGR1 and AGR3, which are commonly found in the ER, AGR2 actively engages in ERS and carries out its designated functions [24]. AGR2 exhibits both intra- and extracellular distribution, and its participation in the ER pathway varies depending on its location. This variance in involvement within the ER pathway leads to diverse intra- and extracellular expressions of AGR2, consequently influencing the progression of the disease.

## 3. Functions of AGR2

The ER primarily functions to regulate protein homeostasis, ensuring the correct folding of newly synthesized proteins. Proper regulation of protein homeostasis is critical in the development and progression of various diseases [25]. AGR2 acts as a folding catalyst within the ER and can fulfill diverse roles due to its multistructural domain structure. Through heterodimerizing disulfide bonds in proteins, AGR2 aids in producing a folded conformation [22]. Compared to other PDI proteins, AGR2 features a single CXXS active domain motif that participates in redox reactions. Notably, in intestinal epithelial cells, the maintenance of the intestinal mucus barrier relies on AGR2, and AGR2 is involved in the maintenance of the mouse intestinal mucus barrier along with MUC2. Park et al. found that the normal integrity of the mouse intestinal mucus barrier is disrupted upon the artificial suppression of AGR2 expression [26], emphasizing its essential role in sustaining the intestinal mucosal barrier [27]. Furthermore, AGR2 is also pivotal in the precise processing of mucin formation, which is vital for intestinal barrier function and acts as a scaffold for antimicrobial factors [28]. The intestinal mucus layer, a key component of the mucosal barrier, plays a central role in maintaining the balance of intestinal flora by nourishing commensal bacteria [28]. Research evidence confirms that imbalances in the gut flora are a significant factor in regulating the progression of cancers, particularly those of the digestive tract, including hepatobiliary and pancreatic cancers [29]. Moreover, there is a connection between the gut microbiota and the clinical response to anti-PD-1 immunotherapy in hepatobiliary tumor patients [30]. Given this evidence, it can be inferred that decreased AGR2 levels compromising mucosal layer integrity may play a significant role in cancer invasion. Conversely, other studies have demonstrated that the overexpression of AGR2 can trigger cancer progression. Despite disparities in findings related to the effects of reducing AGR2, the multifaceted nature of cancer suggests that AGR2 may play varying roles at different expression levels, potentially influencing the entirety of cancer development.

Further investigations have unveiled that the CXXS active structural domain motif is crucial for AGR2’s normal function by engaging in the formation of mixed disulfides with intestinal mucins. This process catalyzes the formation and rearrangement of protein disulfide bonds, a vital protein modification occurring in the ER [31]. Interestingly, the active-site sequence of AGR2 in CXXS lacks cysteine, positioning AGR2 as a late-evolving member of the PDI protein family [32,33]. Conversely, mutations in AGR2 involving cysteine can significantly impact its disulfide exchange activity, potentially affecting functions like intestinal mucin folding and perturbing mucin interactions [22,33]. The accurate folding of disulfide bridges during protein assembly hinges on the balance between the AGR2 monomer and dimerization [25]. Moreover, MUC1 [34] and MUC5AC [35], linked to the CXXS motif in AGR2 proteins, can form mixed disulfides that facilitate the proper functioning of intestinal mucins, collectively contributing to intestinal tract protection.

In addition, researchers have also noted that intrahepatic cholangiocytes typically produce mucin, and AGR2 plays a role in the secretion of protective mucus by intestinal epithelial cells in the intestinal tract. In cases of ICC, AGR2 promotes an increase in extracellular mucin levels. Elevated mucin expression can enhance cancer cell invasion and impact patient prognosis in ICC [36]. The role of AGR2 in elevating mucin levels to influence cancer invasion has been observed in various cancers, including pancreatic [37], colon [38], and breast cancers.

In general, proteins necessitate processing in the ER, with soluble proteins aggregating in the ER through KDEL receptor-mediated Golgi transport [39]. Nonetheless, AGR2 possesses its distinctive ER localization signals. AGR2 features the atypical carboxy-terminal ER retention sequence KTEL, containing the lysine(K)-threonine(T)-glutamic acid(E)-leucine(L) tetrapeptide motif. The KTEL motif in AGR2 can bind to KDEL receptors, thus reinstating the protein’s ER localization function [39]. ER localization is essential for AGR2 to function effectively, with its localization playing a crucial role in cancer cell survival and metastatic pathways [22]. A lack of the KTEL motif results in AGR2 losing its normal function [40]. When other carboxy-terminal sequences, such as KDEL or KSEL, are used instead of KTEL to achieve ER stress, retrograde translocation of proteins in the ER to the extracellular space occurs, ER protein retention occurs [39], and AGR2 loses its normal function, potentially contributing to disease development [22,39]. Apart from its folding role, AGR2, a member of the PDI protein family, serves as a molecular chaperone during ER degradation [32]. Overall, the ER-localized properties of AGR2 are closely tied to the three motifs mentioned previously, collectively driving AGR2’s function. When overexpressed due to ER stress, AGR2 can evoke pro-carcinogenic and inflammatory effects.

A series of in vivo and in vitro experimental studies have demonstrated that AGR2 promotes tumor invasion and metastasis through ERS [41,42]. The main triggers of ERS include oxidation, nutrient deficiency, calcium imbalance, and protein glycosylation. These factors collectively impair the ER’s ability to properly process proteins, leading to the accumulation of misfolded proteins. The excessive buildup of misfolded or unfolded proteins in the ER induces ERS, triggering a UPR [43]. Under normal circumstances, the UPR modulates processes like autophagy, apoptosis, and inflammation in response to ER stress. This adaptive mechanism helps tumor cells evade apoptosis and proliferate excessively. The interplay between the UPR and ER stress plays a crucial role in cancer pathogenesis [44]. A signaling pathway known as IRE1α-XBP1, primarily activated by ERS, represents a key UPR pathway. XBP1s, formed through splicing, facilitates the correct folding and secretion of ER proteins while also promoting intrahepatic lipid synthesis [12,45], and inhibiting this pathway has been shown to alleviate liver fibrosis significantly [43]. A study by Dumartin et al. revealed that AGR2 can influence cancer progression through XBP1s [43]. Silencing AGR2 led to the overexpression of XBP1s, which in turn modulated ERS and maintained ER homeostasis to impede cancer progression [18].

In pancreatic cancer, AGR2 expression is influenced by ERS, impacting pancreatic carcinogenesis, and AGR2 serves as a marker for cancer progenitor cells [18]. Biliary Vater carcinoma, originating from the duodenal mucosa and also known as Vater curvular carcinoma, is a rare subtype of biliary tract cancers [46], Intriguingly, in rare biliary Vater carcinomas, heightened AGR2 expression correlates with increased cancer cell proliferation and invasion capabilities [47].

Understanding the diverse functions of AGR2 underscores its essential role in preserving organismal homeostasis while contributing to cancer invasion through imbalances in self-expression. AGR2 disruption affects normal mucin production, potentially influencing cancer invasion by altering the intestinal mucosal flora. Moreover, AGR2 triggers the UPR through its specific ER function, thereby promoting cancer progression via the UPR pathway. These findings offer valuable insights that may inform future cancer treatment approaches.

## 4. Regulation of AGR2

The expression of AGR2 is intricately regulated by various proteins, predominantly through protein–protein interactions that influence its function [48]. ESE1, identified as a robust activator of the AGR2 promoter in tumors, is regulated by transforming growth factor (TGF)-β [37]. AGR2 is a novel transcriptional target of ESE1, positively correlating with AGR2 to maintain epithelial cellular uniformity. Conversely, TGF-β acts as an antagonist to this process [37], inhibiting AGR2 expression [49]. Additionally, the protein ZEB1 exhibits a negative correlation with AGR2 expression, with increased ZEB1 levels leading to reduced AGR2 expression [50]. AGR2, in turn, facilitates the degradation of ZEB1 mRNA, forming a reciprocal feedback loop crucial during epithelial–mesenchymal transition (EMT) and cancer metastasis [51]. The connection between AGR2 and EMT across various cancer types remains unclear. For instance, in lung adenocarcinoma and pancreatic ductal adenocarcinoma, TGF-β downregulates AGR2 expression to promote EMT [37,52], while in colon and head and neck squamous cell carcinomas, AGR2 overexpression plays a role in EMT promotion [53,54]. Both ZEB1 and TGF-β are involved in inducing EMT, but the role of AGR2 in this process is still unclear. Understanding the interplay among these factors offers potential for further investigation into the mechanisms of cancer development.

Hong et al. identified a factor called KAI1, an anti-cancer factor, which shows a negative correlation with AGR2 expression, with increased AGR2 levels and decreased KAI1 levels associated with advanced tumor stage and grade [55].

During tumor progression, some authors have found that AGR2 and FOXA1 are recognized as prognostic markers during tumor progression, with both proteins interacting and mutually promoting each other at low levels [56]. FOXA1, observed in breast cancer [57] and HCC [58], serves as a transcriptional activator of liver-specific transcripts, stimulating AGR2 expression and fostering HCC recurrence and metastasis [59]. Additionally, Yuan et al. found that FOXA1 can inhibit apoptosis by regulating AGR2 expression, where AGR2 acts as a functional protein of FOXA1 in anti-apoptosis mechanisms [60]. FOXM1, akin to FOXA1, also activates AGR2 [61]. And it was found that miR-212-3p and miR-342-3p modulate AGR2 expression through different pathways [60], with the former influencing AGR2 expression post-transcriptionally via FOXA1 regulation and the latter by directly reducing AGR2 expression levels [62]. The transcription factor Twist1, another regulator of AGR2 [63], can inhibit AGR2 expression in HCC, potentially enhancing cancer patient treatment outcomes [51]. Interestingly, in addition to ER proteins impacting AGR2 secretion, AGR2 expression can be influenced by promoter methylation [64]. Sung et al. [65] and He et al. [66] suggested that hypomethylation of the AGR2 promoter region induces AGR2 expression, potentially promoting invasive cell behaviors under the influence of DNA methylation. Techniques such as methylation-specific PCR have been employed to detect AGR2 expression in cancer cells, revealing significant expression levels in such cells [67].

**Figure 1:**
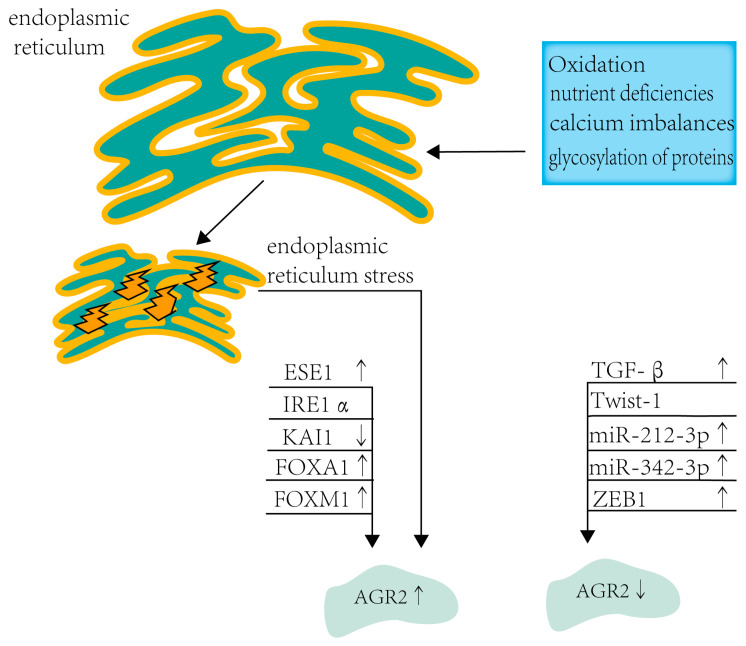
Endoplasmic reticulum stress and multiple proteins regulate AGR2 expression.

Collectively, IRE1α, FOXA1, and other factors have been identified as contributors to the upregulation of AGR2, while molecules such as TGF-β, Twist-1, and others are implicated in its downregulation, collectively influencing AGR2 expression (refer to Figure 1) and impacting disease progression. Understanding the proteins that regulate AGR2 expression is crucial in unraveling the mechanisms of cancer development. Manipulating these processes may serve as a pivotal approach in cancer treatment, leading to the discovery of effective therapeutic strategies.

## 5. Expression of AGR2

AGR2, the most extensively studied member of the PDI family, induces expression through ERS and plays important roles in eukaryotic protein synthesis, transport, and lipid synthesis [68]. The activity of AGR2 can be modulated through various mechanisms [69]. It is noteworthy that the expression of intracellular and extracellular AGR2 (eAGR2) operates relatively independently, each governed by distinct regulatory pathways [21]. A key determinant of this divergence in expression forms lies in the presence of the KTEL structural domain within intracellular AGR2 (iAGR2) [39]. eAGR2 has the capacity to undergo internalization into fibroblasts via endocytosis, subsequently translocating to the nucleus and engaging with β-linker proteins within the cells. This process culminates in the accumulation of β-linker proteins, exerting influence on surrounding fibroblasts, vascular endothelial cells, and the TME, thereby fostering tumor growth [70]. Under normal conditions, AGR2 is expressed in normal epithelial tissues. In cancers, AGR2 is frequently overexpressed, particularly in prostate and gastrointestinal cancers [20,24,71].

### 5.1. eAGR2

eAGR2 exhibits a higher level of aggressiveness compared to iAGR2 [40,72]. Functioning as an AGR2 monomer, eAGR2 operates through the dimerization of residues E60 and C81 [32]. Notably, AGR2 secreted into the TME operates independently of the ER [73]. Conceptualizing eAGR2 as a novel tumor factor disseminated within the TME, it instigates cell proliferation by inhibiting the tumor suppressor P21 (CIP1) [73]. Furthermore, the expression of eAGR2 facilitates cancer invasion through various mechanisms. Firstly, eAGR2 interacts with extracellular matrix (ECM) proteins to enhance the formation of invasive structures [40], establishing itself as a critical pro-carcinogenic regulator of the ECM microenvironment [40]. Secondly, eAGR2 plays a pivotal role in promoting insulin-like growth factor 1-induced EMT, triggering programmed cell death in normal cells [74]. Reports indicate that eAGR2 can infiltrate cells to influence tumor development and invasion from within, leading to the accumulation of β-linker proteins that modulate fibroblasts surrounding tumor cells within the TME [70,72]. AGR2 secreted by ovarian cancer not only sustains tumor growth by regulating the protein cycle and modulating fibroblasts but also acts as a chaperone protein binding to extracellular signaling molecules [75], enhancing its own activity and promoting angiogenesis and tumor growth, including factors like vascular endothelial growth factor (VEGF) and fibroblast growth factor 2 (FGF2) [76]. Furthermore, augmenting signal transduction pathways, eAGR2 ultimately facilitates tumor cell aggregation, migration, and angiogenesis, fostering conditions for tumor growth [76]. eAGR2 expression has been identified in pancreatic cancer [16], gastrointestinal mucinous tumors [77], and prostate cancer models [78], where AGR2 is directly expressed within the TME and secreted by cancer cells [40]. This secretion creates an environment conducive to cancer cell invasion and proliferation [79]. Inhibiting tumor growth can be achieved by targeting the self-dimerization region of AGR2 with monoclonal antibodies to disrupt eAGR2 activity [76]. Given its significant pro-carcinogenic role, particularly in pancreatic cancer, exploring eAGR2 expression could offer valuable insights for combating drug resistance and enhancing treatment efficacy.

### 5.2. iAGR2

iAGR2 resides in the ER and functions as a PDI responsible for catalyzing the proper folding of multiple proteins, thereby facilitating tumor invasion by augmenting ER folding proteins [19]. Typically found in normal cells [40,80], iAGR2 can undergo overexpression in cancer cells [19]. Research has shown that iAGR2 can impact tumor cell proliferation, invasion, and drug resistance. The heightened expression of iAGR2 may serve as a link between ER quality control and tumor advancement, with increased levels contributing to enhanced ER protein homeostasis and stability, subsequently promoting cancer invasiveness in various cancer models [81,82]. Moreover, iAGR2 is capable of transcriptionally regulating the EMT gene pattern through P65, functioning as a facilitator of cancer invasion. Characterized by diminished cell-to-cell adhesion and loss of apical basolateral polarity, EMT represents a pivotal aspect of tumor progression associated with invasive and metastatic behaviors [41,83]. Arumugam et al. have proposed that the lack of cancer-associated iAGR2 significantly diminishes tumor cell proliferation, as demonstrated by a decreased cancer cell proliferative capacity upon silencing AGR2 in cell-conditioned medium stimulation [84]. Additionally, Sicari et al. have identified intracellular cytoplasmic AGR2 within cytoplasmic lysates isolated from mouse and human tumors [67]. When proteins within the ER become destabilized, AGR2 translocates from the ER to the cytoplasmic lysate, potentially interacting with and inhibiting the tumor suppressor P53, thus contributing to cancer progression [82].

The above studies underscore the distinct role played by iAGR2 in cancer invasion, wherein it influences cancer invasion through its enzymatic protein folding function and contributes to the modulation of the EMT gene pattern. Given the prevalence of EMT in various cancer progressions, further investigation into the relationship between iAGR2 overexpression and EMT could offer valuable insights for early-stage disease intervention.

## 6. Association between AGR2 and Hepatobiliary and Pancreatic Cancer

In recent years, there has been increasing evidence suggesting that AGR2 may be involved in tumor development, invasion, and metastasis [16,20,24,56]. The tumor-promoting effects of AGR2 were initially reported in breast cancer by Liu et al. [85] in 2005. And subsequent studies demonstrated its role in promoting tumor progression in cancer cells associated with sex hormone receptors [71,86]. Further research has confirmed that AGR2 can be overexpressed and exert pro-cancer functions in various solid cancers [24], ranging from adenocarcinomas to squamous carcinomas, including breast adenocarcinomas [87], pancreatic adenocarcinomas [88], esophageal adenocarcinomas [89], prostate adenocarcinomas [78], esophageal squamous cell carcinomas [90], and HCC [12].

AGR2 is ubiquitously present in a wide array of cancer types, and investigating its distribution mechanism could potentially enhance cancer detection and treatment strategies. Some studies have suggested that detecting AGR2 in the serum could aid in the identification of pituitary adenomas, breast, ovarian, and gastrointestinal tract cancers [56,69,87,91,92,93,94]. Moreover, investigations into AGR2 expression in urine have shown promising potential for cancer detection [95], particularly in bladder cancers and as a specific marker for prostate cancer cells when secreted into the urine [96]. Though the utility of urine AGR2 as a cancer marker may require further validation with larger sample sizes and stable sample collection methods, it presents a promising avenue for non-invasive, cost-effective diagnostic tests. Additionally, studies have indicated that AGR2 could serve as a predictor of cancer invasion and the effectiveness of immunotherapy in pancreatic and breast cancers [18,52,56,69]. While the role of AGR2 in hepatocellular carcinoma and cholangiocarcinoma remains to be fully elucidated, future research focusing on immunotherapy and exploring the link between AGR2 and these specific cancers is warranted to advance clinical applications in cancer treatment.

### 6.1. AGR2 and Primary Hepatocellular Carcinoma (PHC)

In the progression of HCC, researchers have identified that ERS-induced cell proliferation and metabolic changes are critical at each stage, from steatosis to non-alcoholic steatohepatitis (NASH), hepatic fibrosis, and ultimately leading to HCC [97]. This process is closely associated with the expression of AGR2 [23]. AGR2 is normally expressed in the liver, predominantly in the cholangiocytes of intrahepatic bile ducts [13] and its overexpression resulting from ERS can enhance the uptake of long-chain fatty acids [98],potentially contributing to the development of fatty liver disease. Studies have demonstrated that the UPR triggered by ERS plays a role in promoting HCC development [35]. Specifically, AGR2 induces UPR via ERS, leading to oxidative stress and cell death, directly impacting the progression of NASH [99]. From another perspective, upregulation of UPR markers such as GRP78 and GRP94 is linked to increased cancer aggressiveness and poor prognosis [100,101]. The UPR in immune cells and astrocytes in the vicinity of the HCC tumor microenvironment has been implicated in tumor growth and invasion [102]. The relationship between AGR2 and UPR suggests that AGR2 may act as a central regulator of intestinal cuprocytes’ UPR through IRE1β, potentially modulating the UPR to maintain intestinal homeostasis and influence disease progression [28]. However, in the context of cancer, AGR2’s role in UPR regulation can be invasive. as studies have shown that metastatic HCC cells often exhibit high levels of AGR2 expression, correlating with increased metastatic potential [103]. Additionally, overexpression of AGR2 has been associated with elevated levels of CyclinD1, CDK4, MMP-3, and MMP-4, which are known factors promoting HCC progression [103]. The upregulation of these factors induced by AGR2 may contribute to the invasive nature of HCC cells, as observed in both in vivo and in vitro studies [10]. It is important to acknowledge the impact of AGR2 on promoting molecular pathways that drive HCC progression [104,105].

In 2020, Yang et al. discovered that LINC00460 accelerates HCC progression by increasing AGR2 expression through the sequestration of miR-342-3p [62]. Concurrently, Hong et al. conducted in vivo experiments illustrating that decreased LINC00460 expression boosted miR-342-3p levels via the LINC00460-miR-342-3p-AGR2 pathway. This elevation of miR-342-3p led to reduced AGR2 expression, ultimately demonstrating anti-tumor effects [10]. Yuan et al. revealed that FOXA1 was markedly expressed in HCC tissues, with AGR2 significantly linked to FOXA1 expression. AGR2 functioned as a downstream effector of FOXA1, promoting cancer invasiveness and resulting in a bleak prognosis for HCC patients [60].

Additionally, an investigation was conducted on all studies targeting AGR2 in PHC (refer to Table 1), and the data presented in this table further confirm the significance of AGR2 in PHC diagnosis, immunotherapy, and overcoming drug resistance.

### 6.2. AGR2 and Cholangiocarcinoma (CCA)

Differing opinions exist regarding the expression of AGR2 in normal bile ducts. Some authors suggest that the high epithelial cells covering the bile ducts and gallbladder epithelium express AGR2 [13,110], while others hold a different view [111]. Conversely, AGR2 is overexpressed in cholangiocarcinoma, considered one of the most upregulated metastasis-related genes in highly metastatic cholangiocarcinoma [20]. Similar to liver cancer, the UPR pathway is also present in CCA, promoting cholangiocarcinoma cell survival and evading apoptosis under endoplasmic reticulum stress conditions [20]. The oncogenic AGR2 isoform AGR2vH, generated by splicing, contributes to the metastatic phenotype of cholangiocarcinoma cells [20]. Suwan et al. discovered that the oncogenic AGR2vh isoforms produced by splicing promote ICC cell metastasis and induce ERS with AGR2vh overexpression [112]. Yosudjai et al., through proteomic analysis, suggested the Wnt/β-catenin signaling pathway as a cancer-associated pathway in ICC [113], with AGR2vh promoting ICC cell proliferation through the upregulation of β-catenin. ^125^I can inhibit the invasion of cholangiocarcinoma by suppressing AGR2 expression and modulating related pathways [13,114], although some studies have proposed that AGR2 has limited prognostic value for cholangiocarcinoma and that AGR2 expression decreases with biliary tract cancer progression [111]. However, the evidence from these studies highlights the pro-invasive and metastatic role of AGR2 in cholangiocarcinoma. It is essential to carefully examine the correlation between AGR2 expression and clinicopathological data in a significant patient cohort, potentially positioning AGR2 as a novel therapeutic target in cholangiocarcinoma treatment.

From the evidence presented in these studies, we can infer that AGR2 plays a role in the development and invasion of hepatobiliary and pancreatic cancers through both direct and indirect effects.

### 6.3. AGR2 and Pancreatic Cancer

Pancreatic cancer is no exception to this pattern. Elevated levels of AGR2 resulting from ERS have been discovered in pancreatic fluid from patients with ductal complex and early-stage high-grade pancreatic intraepithelial tumors, indicating a precursor to aggressive pancreatic cancer [18]. Cancer-secreted AGR2 contributes to pancreatic cancer growth and metastasis by promoting endoplasmic reticulum retention and enhancing cancer cell viability. Notably, increased AGR2 expression in pancreatic cancer cells post-chemotherapy treatment has been associated with enhanced cancer cell viability, suggesting a role of AGR2 in the development of chemo-resistance [16]. Ram et al. have shown that AGR2 silencing significantly inhibits cell proliferation, invasion, and survival in vitro, and suppresses tumor growth in vivo, underscoring the importance of AGR2 in pancreatic cancer progression [42].

In certain instances of pancreatic cancer, AGR2 shows promise as a potential tumor cell marker, particularly in ductal cells where it is significantly overexpressed in pancreatic tumors of ductal origin. In contrast, adenophysis cells exhibit minimal AGR2 expression. Scholars have proposed AGR2 as a potential marker for ductal-origin pancreatic cancer due to this expression pattern disparity [52], potentially facilitating enhanced tumor classification for more effective cancer treatment.

A study on mouse pancreatic cancer sequencing results proposed that the growth as well as differentiation of pancreatic ductal adenocarcinoma (PDAC) is regulated by the transcription factor SPDEF, and the PDAC-promoting function regulated by SPDEF can be mediated by AGR2 and ERN2/IRE1β, and the deletion of AGR2 impairs the growth of tumor organoid and classical-like PDAC cells, but has a lesser effect on the cells of basal-like PDAC [115]. In addition, AGR2 is expressed during adeno-ductal metaplasia (ADM) in PDAC [116]. Zaytouni et al. found that AGR2 expression was increased in obese PDAC patients and AKT-driven PDAC patients, the invasiveness of pancreatic cancer cells was proportional to the level of AGR2 expression [42,117,118], and the silencing of AGR2 in human pancreatic cancer cells (MPanc-96) inhibited tumor growth [42]. In contrast, AGR2 in pancreatic neuroendocrine tumor cells plays a role in promoting tumor growth and metastasis through interaction with cancer-associated fibroblasts [119]. Based on these conclusions, understanding the expression of AGR2 may help to stratify PDAC and be more helpful for clinical treatment.

While the exact relationship between pancreatic cancer and AGR2 is still being clarified, overexpression of AGR2 has been linked to increased cancer cell proliferation in various types of pancreatic cancer cells. AGR2 shows potential as a marker for pancreatic cancer, particularly in the context of pancreatic ductal adenocarcinoma. Ongoing research investigating pancreatic cancer treatment strategies involving AGR2 shows promise for future therapeutic approaches.

**Figure 2:**
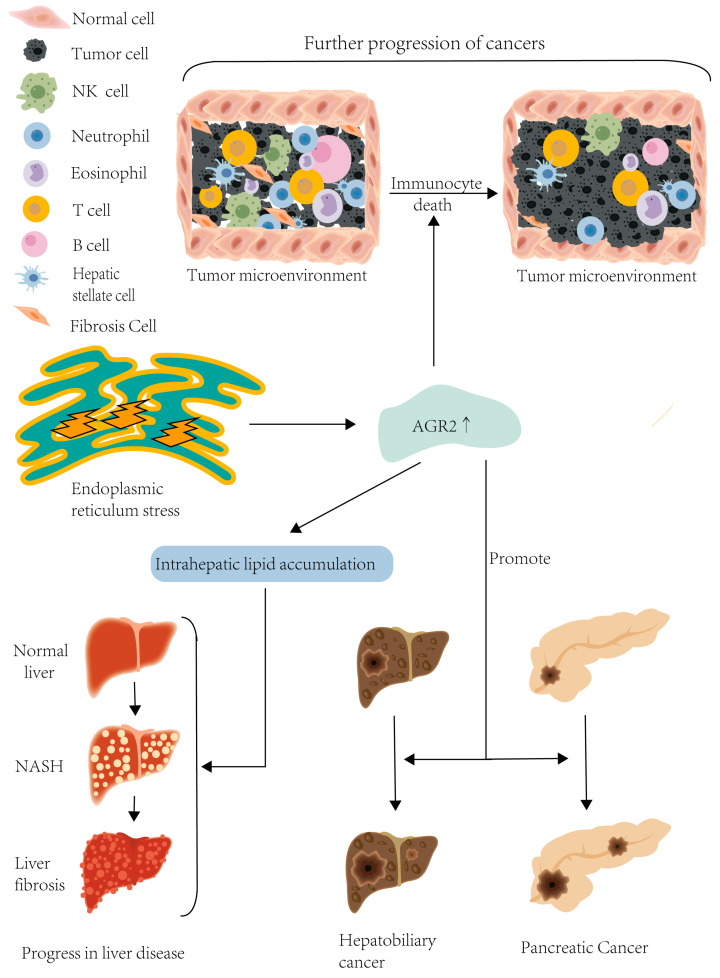
Endoplasmic reticulum stress promotes AGR2 overexpression, which, on the one hand, is involved in the progression and deterioration of liver disease by causing an increase in lipids; on the other hand, AGR2 overexpression induces immune cell death and promotes hepatobiliary and pancreatic Cancer invasion.

In conclusion, though the precise role of AGR2 in cancer is not fully understood, it has been implicated in cancer invasion and metastasis across several cancer types, including hepatocellular carcinoma. Whether acting directly as a pro-oncogenic protein during ERS or indirectly as a downstream target protein, the oncogenic effects of AGR2 cannot be disregarded. Future studies are warranted to delve into the underlying mechanisms of AGR2 and determine its potential therapeutic value in cancer treatment (refer to Figure 2).

## 7. AGR2 and the Clinical Linkage

### 7.1. AGR2-Related Drug Resistance in Cancer

In clinical practice, the emergence of multidrug resistance poses a significant challenge in cancer treatment, particularly in advanced cases of HCC where patients often undergo chemotherapy or targeted therapy [120]. The efficacy of drugs becomes a critical concern in such cases. Research suggests that drug resistance may arise due to conditions of ERS and unfolded protein response, resulting in increased tumor cell proliferation, elevated protein demand, and activation of the UPR within cancer cells. This activation triggers a pro-tumor inflammatory response, further stimulating ERS and ultimately leading to enhanced drug resistance [109].

Several pieces of evidence support this notion. In a recent study in 2020, Cui et al. proposed that sorafenib resistance in HCC is commonly linked to ERS, particularly through its association with cellular autophagy, as shown in a drug sensitization assay involving sorafenib and HCC [121]. ERS can inhibit autophagy to modulate apoptotic signaling, and enhance tolerance to chemotherapeutic agents in HCC cells by regulating intracellular reactive oxygen species levels or directly binding to glutathione [122]. Furthermore, a study in 2023 suggested that under conditions of ERS, sorafenib upregulates AGR2 through post-translational modification, activating IRE1α-XBP1 to jointly regulate ER homeostasis. This process affects HCC sensitivity to sorafenib, with increased AGR2 expression inducing ERS and subsequently elevating drug resistance in cancer cells [12].

Despite advancements in the development of chemotherapeutic and immunotherapeutic agents, drug resistance remains a significant hurdle in achieving optimal treatment outcomes for patients. Recent research efforts have focused on unraveling the mechanisms underlying drug resistance. Analysis of drug-targeted gene pathways has highlighted the close association between AGR2 expression and drug-related signaling pathways such as PTK, JNK, and p38 pathways [64]. AGR2 expression can influence drug resistance in tumor cells via the extracellular signal-regulated kinase/serine–threonine kinase (AKT) pathway [122]. Additionally, the emergence of cancer drug resistance is intricately linked to the UPR triggered by ERS, encompassing signaling pathways that induce resistance in solid tumor cells, including anti-apoptotic signaling, protective autophagy, and non-coding RNAs [123]. Pancreatic cancer [124], non-small cell lung cancer [125], colon cancer [126], and breast cancer [127] exhibit drug resistance via AGR2 involvement. In pancreatic cancer, AGR2 within cancer cells exerts a protective effect post-treatment with gemcitabine chemotherapeutic agents [16]. Studies have indicated that reducing AGR2 expression in the serum can enhance drug efficacy, with initial observations made in colon cancer cases [126]. Moreover, insights into the mechanisms of resistance to tamoxifen [128] and doxorubicin [127] in breast cancer have suggested the presence of AGR2 in the ECM. Proteins within the ECM may be implicated in chemotherapeutic resistance, which can potentially be influenced by inhibiting eAGR2 [21].

These findings collectively demonstrate the close relationship between AGR2 expression and the development of drug resistance, suggesting its potential utility as a biomarker for monitoring treatment efficacy. In conclusion, cancer drug resistance significantly impacts cancer prognosis, highlighting the importance of further investigations into the link between AGR2 and drug resistance. Exploring potential alternative mechanisms influencing drug resistance may open new avenues for innovative cancer combination therapies.

### 7.2. AGR2 and Clinical Treatment

Immunotherapy for cancer is extensively utilized, with the central tenet focusing on the elicitation of antigen-specific T lymphocytes [129]. Dendritic cells featuring AGR2 as a potent antigen are currently harnessed to bolster cancer immunotherapy by activating AGR2-specific T cells to combat cancer cells [130]. Moreover, anti-AGR2 therapy holds significance for cancer cells overexpressing AGR2 [131]. Instances of AGR2 overexpression foster solid tumor invasion, suggesting that anti-AGR2 treatment may prove beneficial across a spectrum of solid tumors [132]. eAGR2 can influence the TME or associated signaling molecules to facilitate cancer invasion, with heightened eAGR2 levels indicative of an unfavorable prognosis for cancer patients.

Notably, immunotherapies targeting anti-eAGR2 antibodies have demonstrated potential applicability in solid tumors [19], with investigations hinting at the potential of eAGR2 targeting to impede cancer cell invasion [133]. A targeted reduction in eAGR2 expression may emerge as a therapeutic avenue for bolstering patient prognosis [134]. As previously highlighted, eAGR2 could impact the TME or engage with signaling molecules, ultimately promoting cancer invasion. Elevated eAGR2 levels have been linked to poorer prognosis among cancer patients. Conversely, research focusing on anti-eAGR2 antibodies suggests that such immunotherapies could hold promise in treating solid tumors, with a supposition that targeting eAGR2 might exert inhibitory effects on cancer invasion. Accordingly, diminishing eAGR2 expression could potentially serve as a therapeutic strategy to ameliorate patient prognosis.

Moreover, AGR2 can be leveraged as a monoclonal antibody, such as 18A4, to enhance the efficacy of other cancer treatments, significantly augmenting the inhibitory impact of drugs on cancer cells [74]. For instance, Roy et al. demonstrated an augmented tumor-killing effect by developing BsAb-AGR2×PD1 for AGR2-positive cancers, intercepting AGR2 paracrine signaling, and rerouting cytotoxic T-cells towards AGR2+ cancer cells to reduce tumor cell viability [135]. These enhanced tumor-killing effects, primarily exploiting AGR2 overexpression in tumor cells, could enhance the efficacy of monoclonal antibody therapy [135]. We posit that AGR2 has already paved the way for novel discoveries regarding its therapeutic role in cancer. While these findings are still in their infancy, they hint at the potential for AGR2 to synergize with various therapeutic approaches.

Furthermore, studies have shown that ERS impacts the functionality of dendritic cells in mouse tumor models. In solid tumors, ERS in dendritic cells and myeloid-derived suppressor cells within the immune system can influence the anti-tumor function of normal T cells. This immunosuppression can be achieved through ERS in T cells, leading to compromised function and apoptosis, ultimately resulting in immunosuppression [123]. At this juncture, dendritic cells may actually promote tumor growth, with the additional contribution of AGR2 to immune evasion by tumor cells [136]. AGR2 in cancer cells plays a key role in modulating communication between immune cells by orchestrating cytokine–chemokine signaling and immune infiltration, thereby influencing the tumor immune microenvironment [64,137].

In a study by Zhang et al., it was found that tumors overexpressing AGR2 exhibit an immunologically “hot” profile, implicating AGR2 in influencing the tumor immune microenvironment. Blocking TGF-β was shown to enhance the anti-tumor response of T cells, suggesting that patients displaying AGR2 overexpression might benefit from a combination therapy involving immunosuppressants and TGF-β blockers [64]. Furthermore, Bian et al. increased the immune sensitivity of HCC cells by investigating the effect of AGR2-derived peptide P1 on major histocompatibility complex class 1-associated chain A/B and NK cells in HCC cells, which can activate the p38 MAPK cell signaling pathway [108].

In HCC, there is a substance named miR-212-3p, which can upregulate AGR2 by regulating FOXA1 to promote the proliferation of HCC [60]. Regarding this type of pathway of action, the authors believe that it can be used as an ideal target for the purpose of HCC immunotherapy. However, such immunotherapy relies on the upregulation of AGR2 expression. Another substance, increased LINC00460 expression, can inhibit miR-342-3p, which can lead to the upregulation of AGR2, thus promoting cancer invasion [62]. Both of these pathways could serve as ideal targets for HCC immunotherapy.

The IRE1α-XBP1 signaling pathway regulates ERS in HCC, promoting the expression of AGR2 [12]. This pathway also triggers macrophage UPR activation, leading to the production of cytokines like IL-6, IL-23, and TNF-α [138,139]. Researchers have observed that macrophages can mirror and amplify the inflammatory effects of tumor cells [138]. Cytokines such as IL-4, IL-6, and IL-10 can in turn reactivate the IRE1α-XBP1 signaling pathway, establishing a cycle of inflammation [140]. Aberrant activation of IRE1α-XBP1 influences T cell differentiation in the tumor microenvironment (TME), consequently promoting tumor cell expression [12]. During the progression of pancreatic cancer, intriguing findings have emerged regarding the interplay between TGF-β-induced EMT and the ESE1-AGR2 axis. Specifically, this axis appears to counteract TGF-β-induced EMT in pancreatic cancer [37]. Interestingly, both ESE1 and AGR2 are also present in HCC [141]. Although the precise mechanism remains unknown, we can speculate that AGR2 and ESE1 may play a role in antagonizing EMT. In the realm of intrahepatic and extrahepatic cholangiocarcinoma, AGR2 immunotherapy is still in the exploratory phase. Despite not being universally detected in most HCC cases, the collaboration of proteins with AGR2 that foster HCC progression should not be discounted, especially in the context of immunotherapy.

In pancreatic cancer, AGR2 functions as a novel surface antigen that facilitates cancer invasion. Closed monoclonal antibodies against AGR2 and C4.4A developed in mouse studies have shown reduced cancer invasiveness [84]. miR-129, which targets the FOXA2-AGR2 pathway, has been found to inhibit pancreatic cancer proliferation [142]. Various researchers have proposed that anti-AGR2 approaches have the potential for pancreatic cancer immunotherapy. Knocking down AGR2 expression has shown tumor-suppressive effects [19,42,142]. The use of eugenic acid in pancreatic cancer reduced AGR2 expression in cancer cells. Additionally, combining AGR2 antibodies with gemcitabine in mouse-transplanted human pancreatic cancers has demonstrated strong immunotherapeutic capabilities, exceeding gemcitabine treatment alone [19]. While different therapeutic strategies have been explored, such as investigating effective upstream factors and targeted combination antibody therapy, further evaluation in clinical settings is important for validation.

Research on immunotherapeutic targets for various cancers has shown promising results. Early in vitro studies using dendritic cells indicated that introducing the AGR2 gene into these cells resulted in beneficial effects in targeting colorectal cancer (CRC). This approach led to increased expression of HLA-DR, CD80, and CD86, along with elevated levels of AGR2-specific cytotoxic T-lymphocytes. These findings suggest that AGR2 may serve as a potential immunotherapeutic antigen against CRC. AGR2 has consistently emerged as a potential therapeutic target in colon cancer from various perspectives [15,126,143]. AGR2 is overexpressed in esophageal squamous cell carcinoma and that AGR2 expression may serve as a biomarker for predicting the response to treatment in esophageal squamous cell carcinoma and as a potential therapeutic target for patients with P53 wild-type esophageal squamous cell carcinoma [90,144]. Targeting the link between ovarian cancer [145], esophageal squamous cell carcinoma [90], and other cancers with AGR2 immunity, we can hypothesize that AGR2 influences the immune microenvironment of patients, suggesting a potential undiscovered link between the two that may impact patient prognosis.

Overall, immunotherapy targeting AGR2 expression in cancer is still in the preliminary stages and has not yet been incorporated into clinical practice. Extensive clinical trials are essential for further exploration. Moving forward, it is important to investigate both the upstream and downstream mechanisms of AGR2. By identifying drugs that specifically target AGR2 or inhibit endoplasmic reticulum stress, their efficacy in reversing cancer or increasing cancer cell susceptibility can be enhanced. Selectively targeting AGR2 expression in cancer may provide survival advantages for patients with tumors. This ongoing research holds promise for further advancements in immunotherapy for hepatobiliary and pancreatic cancer.

### 7.3. Possible Use of AGR2 as a Tumor Prognostic Marker

Immunotherapy is continuously evolving and has emerged as a crucial therapeutic avenue for cancer patients. However, not all patients respond favorably to these treatments, highlighting the urgent need to identify suitable biomarkers for cancer treatment. Recent reports have suggested that AGR2 could serve as an effective biomarker for immunotherapy.

AGR2 overexpression is closely linked to tumor prognosis, often indicating a poor prognosis [146]. The study corroborates this observation. Investigating the interplay between AGR2 and FOXA1, researchers found that concurrent overexpression of both proteins promotes HCC progression. Subsequent exploration of AGR2’s upstream influences revealed that in the LINC00460-miR-342-3p-AGR2 axis, AGR2 overexpression, in the presence of upstream factors, further drives HCC progression [10,62]. Additionally, studies have implicated AGR2 in promoting HCC metastasis, although the precise metastatic mechanism remains unclear [103]. In contrast, AGR3 has been identified as a discriminator between hepatocellular carcinoma and cholangiocarcinoma [147]. Turning our attention to pancreatic cancer, clinical investigations have underscored the significance of AGR2. Notably, downregulation of AGR2 expression does not induce EMT or lead to a more aggressive phenotype, positively impacting patient prognosis [148]. These findings suggest that AGR2 may serve as a valuable molecular therapeutic target in pancreatic cancer and a favorable prognostic marker.

Furthermore, studies have highlighted the role of AGR2 in colorectal cancer liver metastasis, where AGR2 secreted by tumor neutrophils contributes to metastatic HCC through the AGR2-CD98HC-xCT axis [15]. In breast cancer, AGR2 has been identified as a marker for tumor invasion, metastasis, and prognosis [87]. The expression of AGR2 in the TME has been associated with the infiltration of tumor immune cells (such as memory B cells, CD8+ T cells) and better prognosis [148]. The TME comprises a diverse immune component, and AGR2 expression can influence the production of various immune cells involved in tumor eradication. Consequently, it is reasonable to speculate that AGR2 somehow modifies the immune microenvironment in patients, or at the very least, there exists a close association between the two. The former has the potential to impact the cellular components of the latter, ultimately influencing the outcome of immunotherapy [73,149].

Finally, despite the advancements in cancer immunotherapy, challenges such as drug resistance and suboptimal treatment outcomes persist [150]. Identifying immunotherapeutic markers like AGR2 can help predict the therapeutic efficacy of drugs and improve patient prognosis. Further research in this field is essential to enhance the understanding of the complex interactions between AGR2, the immune microenvironment, and treatment outcomes in cancer patients.

## 8. Conclusions

The current research findings indicate that AGR2 overexpression adversely affects patient prognosis. Both eAGR2 and iAGR2 play crucial roles in cancer progression. Moreover, the correlation of AGR2 function with cancers such as hepatobiliary and pancreatic malignancies offers valuable insights for cancer treatment. AGR2 shows promise as a prognostic marker by influencing the tumor microenvironment and immune infiltration, thereby impacting drug resistance and patient outcomes.

In summary, AGR2 holds a central role in cancer progression and immunotherapy. While there has been some advancement in AGR2-related immunotherapy, there are currently no clinical drugs specifically targeting AGR2 for patients. Furthermore, certain cancers have been studied with limited sample sizes. Comprehensive research is essential to explore AGR2’s potential in precise drug screening, immune modulation, and resistance mitigation. The implementation of these studies will necessitate extensive in vitro and in vivo experiments, presenting a substantial challenge.

## Figures and Tables

**Table 1:** Recent research on AGR2 in PHC.

Research Subject	Time	Conclusion	Reference
FLC	2009	AGR2 is overexpressed in hepatocellular carcinoma	[14]
ICC	2011	AGR2 is overexpressed in hepatocellular carcinoma	[13]
HCC	2012	AGR2 promotes HCC invasive metastasis	[103]
HCC	2015	AGR2 promotes HCC invasive metastasis	[22]
HCC	2016	AGR2 is overexpressed in hepatocellular carcinoma	[97]
HCC	2018	AGR2 promotes HCC invasive metastasis	[106]
HCC	2019	AGR2 is overexpressed in hepatocellular carcinoma	[107]
HCC	2020	AGR2 promotes HCC invasive metastasis	[108]
ICC	2020	AGR2 promotes HCC invasive metastasis	[20]
HCC	2020	AGR2 promotes HCC invasive metastasis	[60]
HCC	2022	Therapeutic contribution of AGR2 to hepatocellular carcinoma	[109]
PHC	2023	ERS promotes liver disease progression	[43]
HCC	2023	Therapeutic contribution of AGR2 to hepatocellular carcinoma	[12]

## Data Availability

No new data were created or analyzed in this study. Data sharing is not applicable to this article.
